# Facial fractures: classification and highlights for a useful report

**DOI:** 10.1186/s13244-020-00847-w

**Published:** 2020-03-19

**Authors:** Eva Gómez Roselló, Ana M. Quiles Granado, Miquel Artajona Garcia, Sergi Juanpere Martí, Gemma Laguillo Sala, Briggitte Beltrán Mármol, Salvador Pedraza Gutiérrez

**Affiliations:** 1 Radiology Department, Hospital Josep Trueta, Avda França SN, 17001 Girona, Spain; 2 Maxillofacial Surgery Department, Hospital Josep Trueta, Girona, Spain

**Keywords:** Facial trauma, Le Fort, Naso-orbito-ethmoid fractures, Zygomaticomaxillary complex fracture, Mandibular fractures

## Abstract

In patients with facial trauma, multidetector computed tomography is the first-choice imaging test because it can detect and characterize even small fractures and their associated complications quickly and accurately. It has helped clinical management and surgical planning, so radiologists must communicate their findings to surgeons effectively. In Le Fort fractures, there is a breach between the pterygoid plates and the posterior maxilla. These fractures are classified in three basic patterns that can be combined and associated with various complications. Conceptualized when low-speed trauma was predominant, the Le Fort classification system has become less relevant giving more importance on maxillary occlusion-bearing segments. The classification of naso-orbito-ethmoid depends on the extent of injury to the attachment of the medial canthal tendon, with possible complications like nasofrontal duct disruption. Displaced fractures of the zygomaticomaxillary complex often widen the angle of the lateral orbital wall, resulting in increased orbital volume and sometimes in enophthalmos. Severe comminution or angulation can lead to wide surgical exposure. In orbital fractures, entrapment of the inferior rectus muscles can lead to diplopia, so it is important to assess its positioning and morphology. Orbital fractures can also result in injuries to the globe or infraorbital nerve. Frontal sinus fractures that extend through the posterior sinus wall can create a communication with the anterior cranial fossa resulting in leakage of cerebrospinal fluid, intracranial bleeding. It is essential to categorize fracture patterns and highlight features that may affect fracture management in radiology reports of facial trauma.

## Key points

- Radiologists should know anatomical classifications expressed as struts/buttresses and thirds as is the nomenclature used by many surgeons.
- Merely listing fractured bones in the report is useless for surgeons.
- Reports should focus on critical structures affected because of possible complications.
- Displacement and comminution determine the need for surgery, bone grafting, etc.

## Background

Many patients seen in emergency departments have facial trauma. In these patients, major findings may go undetected due to multiple trauma, clinicians’ inability to perform a thorough physical examination, patients’ inability to cooperate, and pronounced facial swelling; thus, facial injuries can be challenging for trauma surgeons [1].

Most patients with facial trauma are male (56.8–92.8%) [2–8], and the mean age in reported series ranges from 24.6 to 51.0 years [4, 9–12]. The most common causes of facial injury are assault (44–61%), traffic accidents (15.8%), and falls (15%) [1, 13, 14]. In patients who require surgery, the most commonly fractured bone is the mandible (41.6–75.2%) [1, 13, 15]. The second and third most commonly fractured bones vary with the series, being the maxilla and orbit (39.8% each) in one series [1] but the malar bone (15.2%) and maxilla (6.4%) in another [15]. In all emergency department patients (those who require surgery and those who do not), closed nasal bone fractures are the most common, being found in 30.1% to 55.8% [1, 13, 14].

Although some authors affirm that appropriate physical examination of the face reliably rules out fractures in some patients (as low impact trauma ones) and some clinical variables are associated with facial fractures [16], physical examination alone cannot classify facial injuries. In other patients like such with polytrauma is widely known that even that physical exam does not rule out fractures because of distracting injuries, obtundation, or facial swelling [17]. Thus, imaging is critical for surgeons to understand which anatomic structures are involved so they can plan the surgical approach and intraoperative technique [18, 19].

It is essential to know the typical patterns and classifications of facial fractures, including those of the zygomaticomaxillary complex and naso-orbito-ethmoidal complex, because each pattern is often associated with particular functional and esthetic complications [20]. There are also specific terms to classify the location of mandibular and orbital fractures. Radiologists’ interpretations of CT scans are important for planning surgery in patients with facial trauma. To ensure efficient communication with surgeons, radiology reports should use the anatomic descriptors and classification schemes that surgeons are familiar with; otherwise, surgeons may choose to rely on their own interpretation of the images [1]. However, an important obstacle in the management of facial trauma is that only low level evidence supports current recommendations [21].

## Techniques of study

Thanks to its widespread availability, computed tomography (CT) is the reference standard for facial imaging [18]. In patients with multiple trauma, facial CT can be easily incorporated into contrast-enhanced whole-body CT protocols, whereas in patients with low-impact trauma, CT images of the face can be acquired together with unenhanced CT studies of the brain or cervical spine [22, 23].

Moreover, even in trauma traditionally diagnosed with plain-film radiography, such as mandibular fractures, CT is more sensitive [24]. Surgeons often use three-dimensional images for planning operations to restore alignment and correct cosmetic deformities, and occasionally these can also be useful for radiologists because they provide a summary view of complex midface fractures.

Between the emerging advances in CT imaging, it stands out the growing use of cone-beam CT: it can make diagnosis of low-energy mandible fractures in walk in clinics, is also used intraoperatively, and has excellent spatial resolution and low radiation dose. As limitations of the technique, the patient must be upright for most units and contrast is not used, so it is not useful in patients with polytrauma [25].

Some techniques are being used in whole-body computed tomography algorithm to decrease the radiation dosis as the Adaptive Statistical Iterative Reconstruction V (ASiR-V). In a recient study by Elmokadem et al., it was proved that biphasic computed tomography protocol reduced radiation dose with maintenance of diagnostic accuracy and image quality after implementing ASiR-V algorithm [26].

As the advantages of CT are so evident in facial trauma classically, there has been a scarce role for MR imaging even advanced techniques like diffusion-weighted imaging (DWI) have not added new utilties for facial trauma. In case of orbit, nasal, paranasal, and skull base lesions, MR evaluation with DWI and ADC levels in is a non-invasive imaging parameter that can help mainly to discriminate between benign and malignant causes [27–29].

## Facial anatomy and landmarks

Five paired and four facial unpaired bones fit together to form the facial skeleton, so it can be cumbersome to characterize facial fractures according to the bones involved. Thus, it is more useful for radiologists to describe how facial fractures relate to structures like orbits or sinuses.

**Fig. 1:**
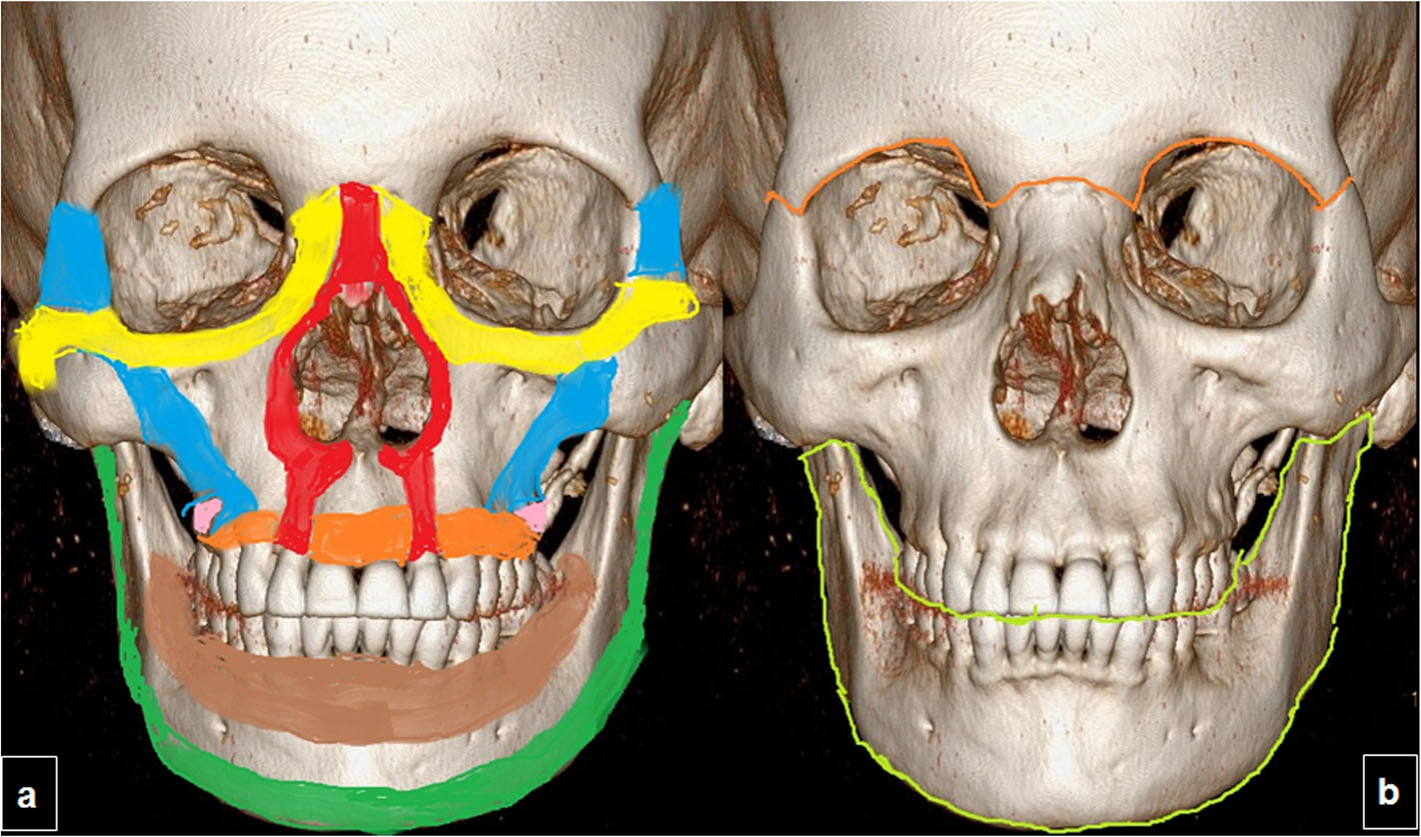
Facial anatomy. **a** System of facial struts/buttresses. Three-dimensional CT images of an adult skull in frontal view with color overlays. The horizontal buttresses are the upper transverse maxillary (yellow), lower transverse maxillary (orange), upper transverse mandibular (brown), and lower transverse mandibular (green) buttresses. The vertical buttresses are the medial maxillary (red), lateral maxillary (blue), posterior maxillary (pink), and posterior mandibular (green) buttresses. **b** System of facial partitions. Three-dimensional CT image of an adult skull with color overlays shows partition of facial anatomy into upper (outlined in orange), middle, and lower (outlined in green) thirds, the system used by otolaryngologists to describe locations of fracture

It is useful to simplify the skeletal structure into four pairs of horizontal and four pairs of vertical struts or buttresses, because this conceptual view emphasizes the functional relations between the different bones in the facial anatomy (Fig. 1). Because the bone in the facial buttresses are thicker than in the rest of the face, these structures form a strong framework that protects the teeth, nasal cavity, sinuses, and contents of the orbits. Damage to the buttresses can modify the configuration of the face and perturb function, and surgical fixation can be required to restore morphology and function. The vertically oriented buttresses connect the bones of the face to the base of the skull. The four vertical buttresses are the medial maxillary buttress, lateral maxillary buttress, posterior maxillary buttress, and posterior mandibular buttress. The four horizontal buttresses are the upper transverse maxillary buttress, lower transverse maxillary buttress, upper transverse mandibular buttress, and lower transverse mandibular buttress [30]; the frontal bar could be included as a fifth buttress [31]. Nowadays, buttress system is “falling out of fashion” among many plastic reconstructive surgeons. Surgeons still use some of the buttress terminology because these are structures with enough bone stock to place small plates and screws.

Furthermore, otolaryngologists normally use a different classification scheme, which divides the facial skeleton into upper, middle, and lower thirds (Fig. 1), and this scheme can also be helpful for planning the surgical approach

It is essential to report the involvement of critical structures or landmarks, where different patterns of fracture could determine major complications (Table 1).

**Table 1:** Facial fractures complications

Affected structures	Complications
Intraorbital contents	Blindness, ophthalmoplegia and diplopia, increased orbital volume with exophthalmos
Nerve foramina	Orbital apex (CN I) → unilateral blindness Superior orbital fissure-(CN III, CN IV, CN V1, and CN VI) → ophthalmoplegia, diplopia, ptosis Mandibular canal (branch of CN V3) → anesthesia of the ipsilateral lower lip, chin, anterior tongue, and mandibular teeth.
Temporalis muscle impingement	Trismus
Teeth	Dental fracture, avulsion, devitalization, malocclusion, soft-tissue infection, airway aspiration
Drainage canals impairment	Frontal recess, sphenoethmoidal recess or ostiomeatal complex → mucocele Lacrimal duct → dacryocystitis
Medial canthal tendon	Telecanthus
Cribriform plate	Leakage of cerebrospinal fluid
Multiple middle face fractures, condylar fractures	Blunt carotid artery injury
Posterior extension	Blunt carotid artery injury, skull base nerve foramina affectation

*CN* cranial nerve

**Fig. 2:**
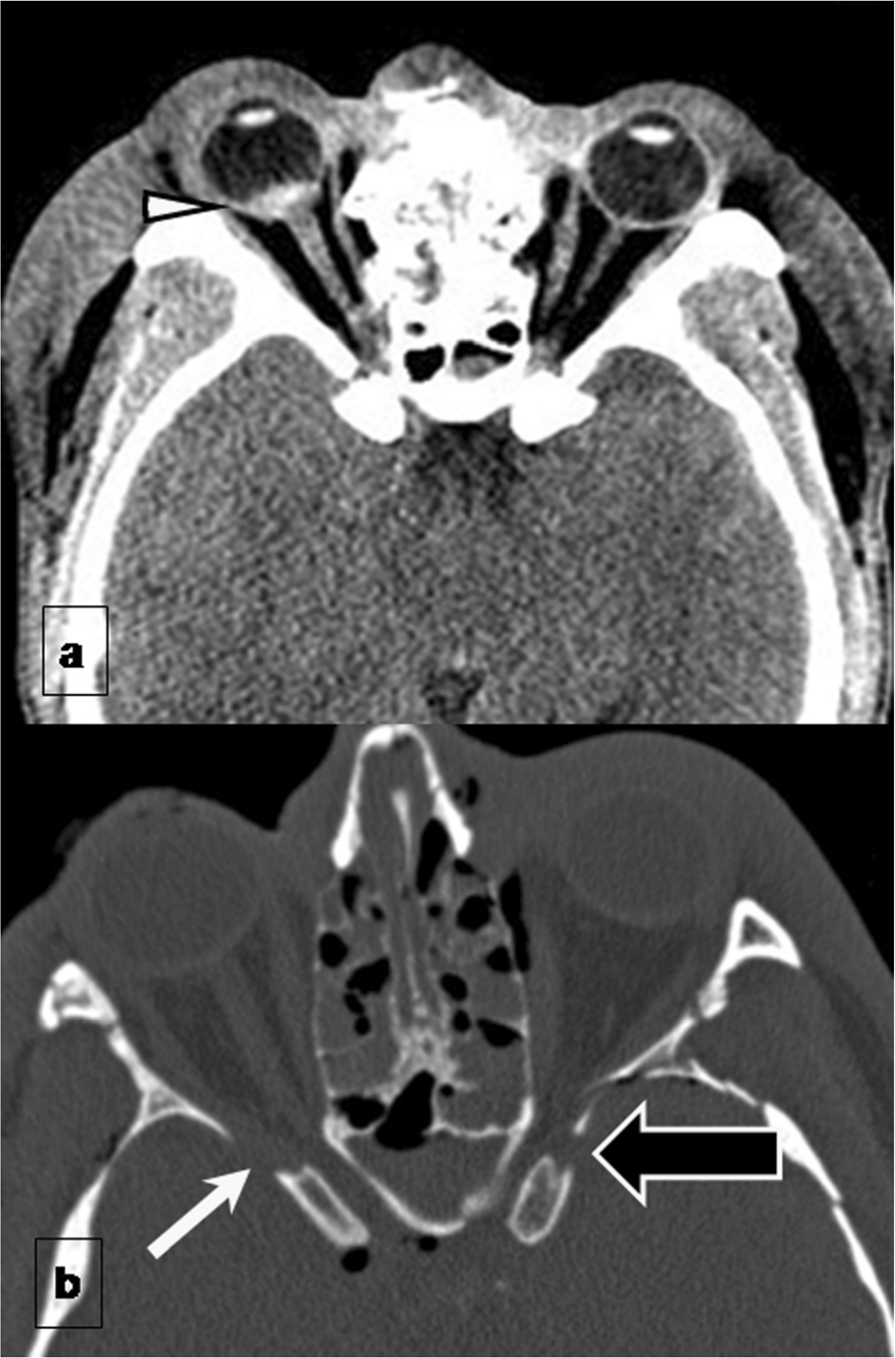
Complications of facial fractures. **a** Intraorbital complications. Axial unenhanced CT at the level of the mid-orbit shows a hematoma of the posterior pole of the globe affecting optic nerve papilla (arrowhead) in a naso-orbito-ethmoid fracture (not shown). The patient developed traumatic optic neuropathy with right blindness. **b** Neural foramina complications: superior orbital syndrome. CT shows left superior orbital fissure narrowing (black arrow) with respect to the normal right side (white arrow) by Le Fort III fracture with external wall displacement that can affect CN III, CN IV, CN V1, and CN VI

Facial fractures often involve risks to intraorbital contents. The inferior rectus muscle can herniate through a fracture, or it can be torn, avulsed from the globe, or entrapped, leading to ophthalmoplegia and diplopia. Rupture of the globe may result in blindness; on CT, a ruptured globe is seen as the “flat tire” sign (deformity of the globe) or an optic nerve lesion (Fig. 2a) [31]. Traumatic optic neuropathy (TON) can be confirmed using MRI as hyperintensity of the optic nerve due to diffusion restriction can serve as a specific imaging marker and when paired with reduced ADC values, an important surrogate for visual acuity [32].

Fractures extending to foramina or to the canals which nerves pass through can damage nerves. When the orbital apex is involved, the optic nerve (CN II) can be damaged, resulting in unilateral blindness. When the superior orbital fissure (Fig. 2b) is involved, CN III, CN IV, CN V1, and CN VI can be damaged, causing ophthalmoplegia or diplopia and ptosis. This constellation of findings can be referred by the blanket term of superior orbital fissure syndrome (SOFS).

**Fig. 3:**
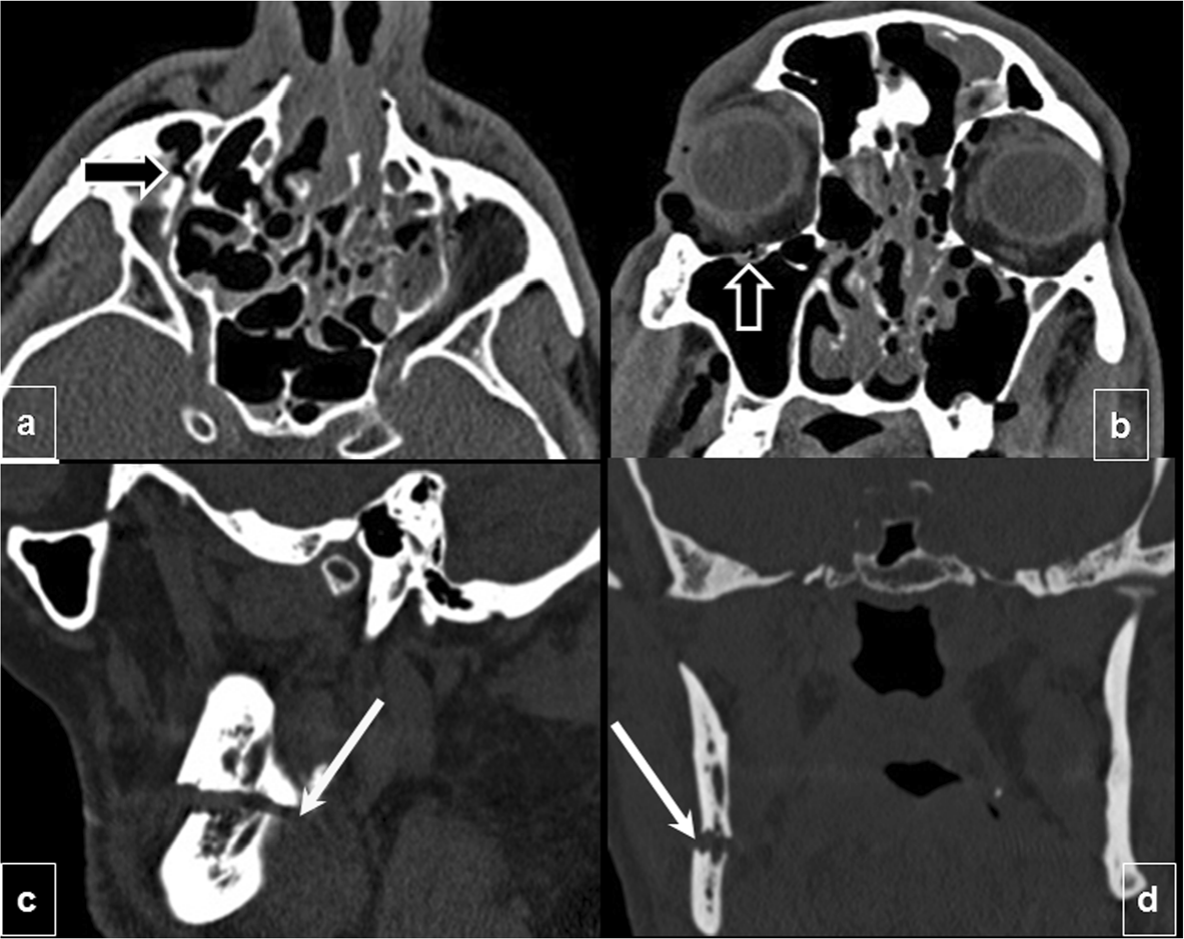
Neural foramina complications. Infraorbital canal injury: (**a**) Axial and (**b**) coronal CT images show a Le Fort II fracture passing through the infraorbital canal, which can affect the infraorbital nerve (terminal branch of CN V2) (black arrow). **c**, **d** Mandibular canal injury: (**c**) Sagittal and (**d**) coronal views of a unifocal mandibular fracture passing through the mandibular canal with mild displacement (white arrows) that may damage the inferior alveolar nerve (a branch of CN V3)

When the infraorbital canal is involved, CN V2, a terminal branch of the maxillary division of the trigeminal nerve (Fig. 3a and b) that traverses the orbital floor within the infraorbital nerve can be damaged, resulting in temporary or permanent hypoesthesia of the ipsilateral cheek and maxillary gingiva. Fractures through the mandibular canal (Fig. 3c, d) may damage the inferior alveolar nerve (a branch of CN V3), resulting in loss of sensation in the lower lip, chin, anterior tongue, and mandibular teeth on the injured side.

If the temporalis muscle is injured or becomes impinged in the infratemporal fossa, patients can develop trismus.

**Fig. 4:**
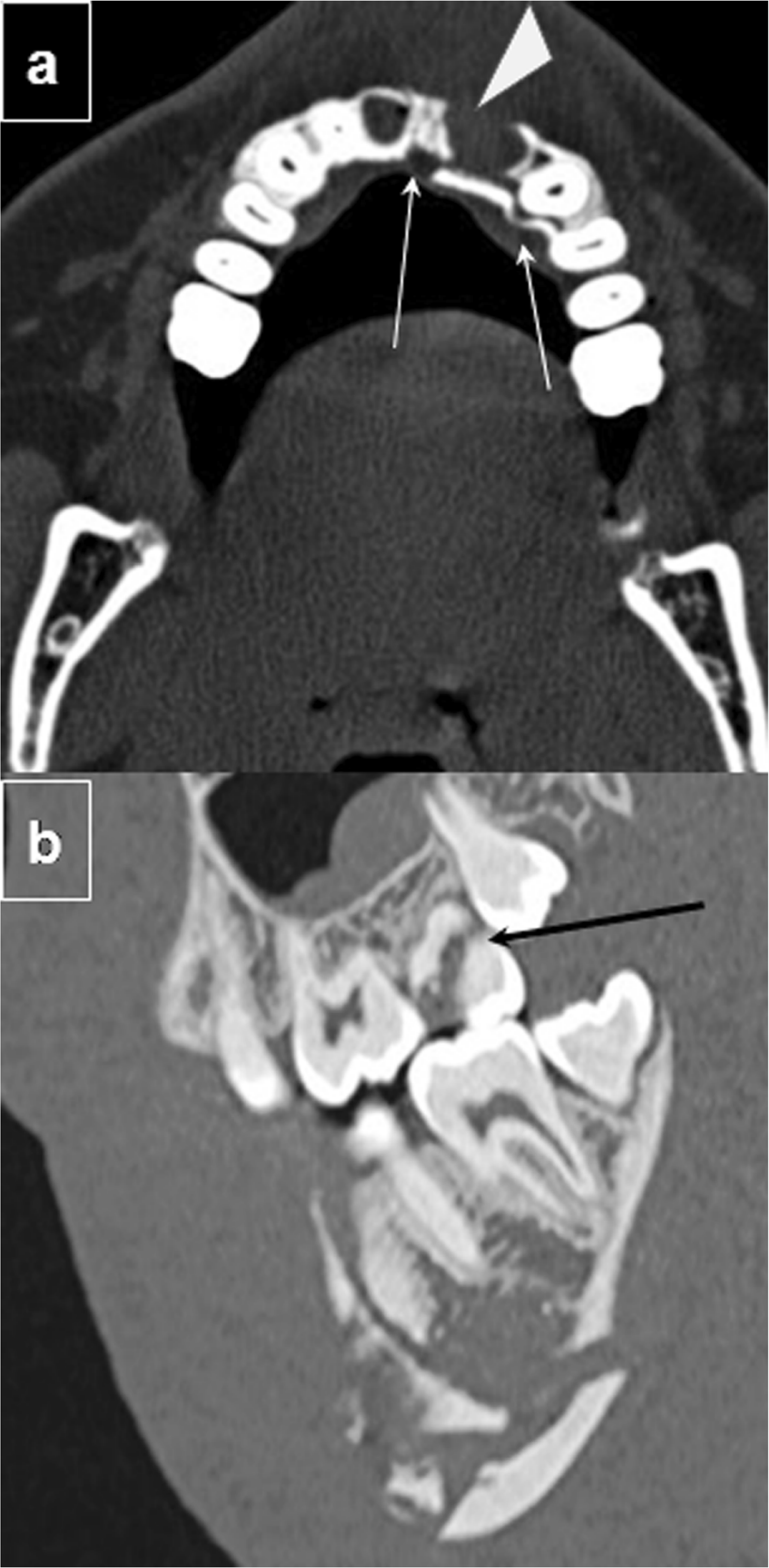
Dental complications. **a** Axial CT image shows a fracture of the upper alveolar ridge (thin white arrows) with avulsion of the left incisors (arrowhead). **b** Sagittal unenhanced CT image at the level of mandibular body demonstrates a molar crown fracture (black arrow) associated with a mandibular fracture

Alveolar bone fractures can result in dental complications such as fracture, avulsion, devitalization, and/or malocclusion of teeth; furthermore, germs from the mouth can invade damaged soft tissues adjoining the fracture, leading to infection (Fig. 4a, b).

**Fig. 5:**
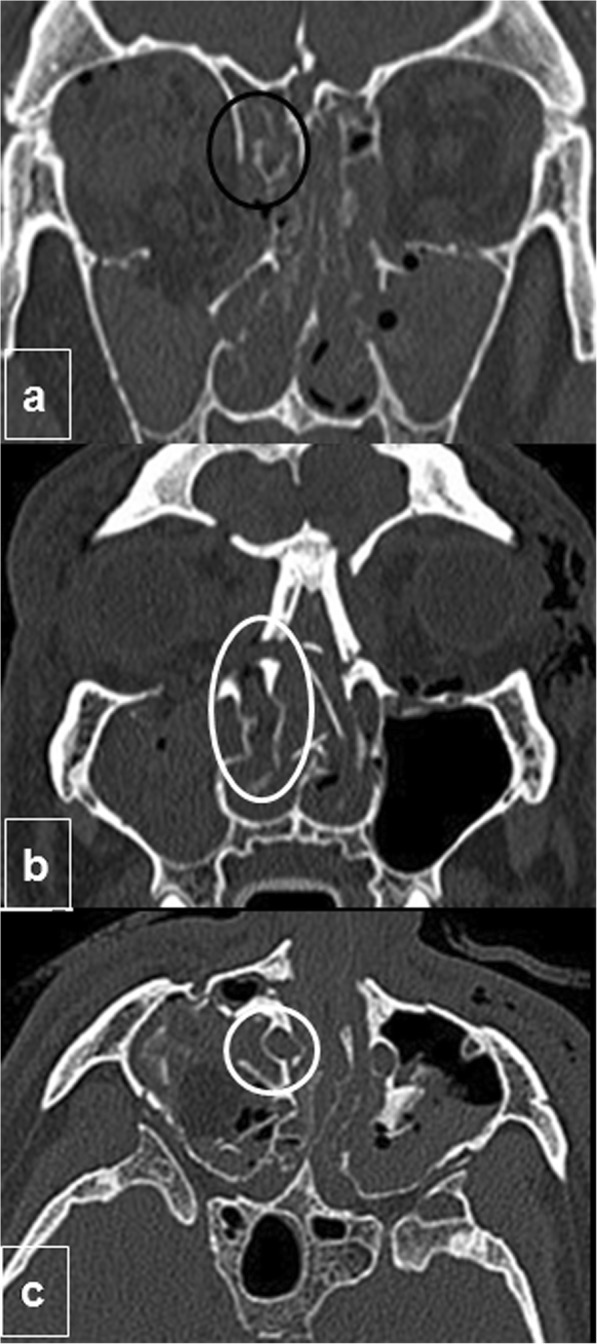
Complications involving drainage canals. **a** Coronal CT image shows frontal recess involvement by fracture (black circle). **b** Coronal and (**c**) axial CT images at the level of the maxillary sinus showing extension of naso-orbito-ethmoidal complex fractures through both nasolacrimal ducts on the right side (circled in white)

Predominantly medial fractures can damage drainage canals and should be surgically repaired. When the frontal recess (Fig. 5a), sphenoethmoidal recess, or ostiomeatal complex are affected, sinus drainage can be obstructed, sometimes resulting in a mucocele. Damage to the lacrimal duct and sac (Fig. 5b, c) can cause chronic epiphora or even dacryocystitis but its obstruction is not really a diagnosis made by CT imaging: severe disorganization of bone in this region does not necessarily predict NLD obstruction and bony alignment does not exclude it, since occlusion can also occur from scar [30]. Medial canthal tendon lesion in the lacrimal fossa can lead to telecanthus with considerable deformity.

**Fig. 6:**
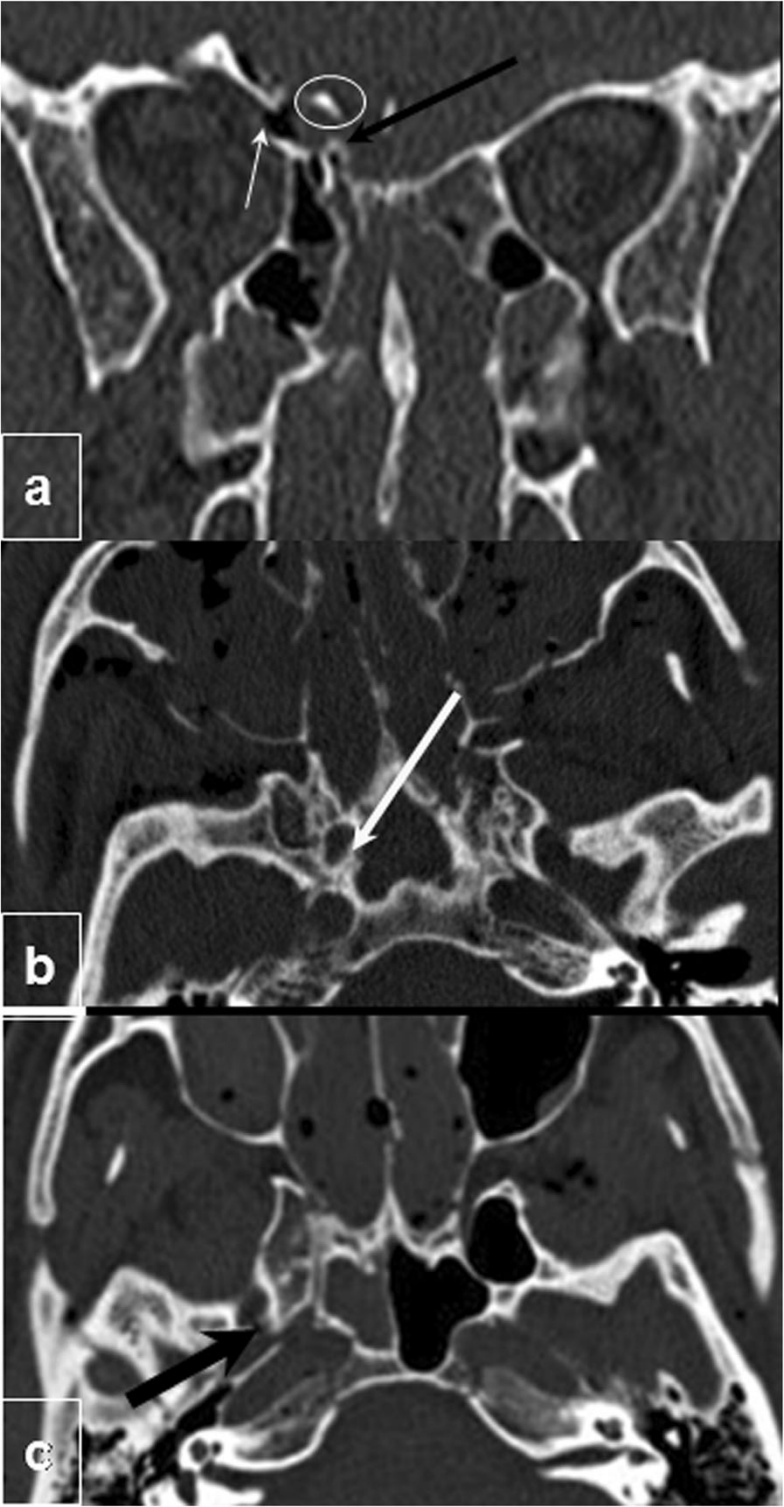
Skull base extension. **a** Anterior cranial fossa: coronal CT image shows a fracture extending to the lamina cribrosa, communicating the ethmoid cells with the anterior fossa of the cranium on the right side (black arrow) and spanning the orbital roof (white arrow). There is a small intracranial bone fragment (circled). This fracture may cause cerebrospinal fluid rhinorrhea and even infectious intracranial complications. **b**, **c** Middle cranial fossa axial CT images from two different patients show extension of the fracture into the sphenoid bone and skull base affecting the carotid canal (white arrow in **b**) and foramen ovale (black arrow in **c**) with possible injury to carotid arteries and cranial nerves

Fractures extending superiorly to the cribriform plate can cause a tear in the underlying dura, allowing cerebrospinal fluid to leak into the paranasal sinuses and nasal cavity (Fig. 6a). Those extending to the paranasal sinuses can create a communication with the anterior cranial fossa, allowing bacteria to enter this normally sterile space and causing infection [31].

Subcondylar fractures and some patterns of facial fracture (including bilateral fractures in any facial third and complex midface fractures, Le Fort I), especially in association with skull base fractures, confer increased risk of blunt carotid artery injury [33]. In addition to carotid artery injury, fractures extending posteriorly into the sphenoid bone and skull base can also affect other foramina, such as the foramen ovale (Fig. 6b, c), in which CN V3 is located [34].

## Patterns and classifications

To ensure effective communications with surgeons, it is extremely important for radiologists to describe patterns [1, 30].

The AO group has proposed a system for classifying craniomaxillofacial fractures in adults (AOCMF) in which anatomic modules are arranged into a hierarchy with three levels of precision to describe these injuries in terms of complexity and details. Level 1 is the most basic; it conveys only whether fractures are present in four anatomical units: the mandible, midface, skull base, and cranial vault. Level 2 describes the location of the fractures in detail within specific regions of the mandible, central and lateral midface, internal orbit, endocranial and exocranial skull base, and cranial vault. Level 3 reports even greater detail about the location of the injury, focusing on morphology (fragmentation, displacement, and bone defects) within specific subregions [35]. The AO classification is not widely adopted for now but it is a promising research tool for the future.

Multidetector CT’s exquisite depiction of bone has enabled the development of new subunit-specific principles for the management of midfacial fracture that are supplanting the older, more general Le Fort classification system, which does not adequately reflect the complexity of the individual components of the midfacial region [36]. Nevertheless, the Le Fort classification remains relevant because it is well known and is still widely used in clinical practice.

The following discussion of fracture patterns first focuses on those involving more buttresses and then focuses on those that involve fewer buttresses (e.g., orbital “blowout” or mandibular fractures). It is important to keep in mind that various patterns often coexist in the same patient (Table 2).

**Table 2:** Patterns and classifications of facial fractures

Multiple buttresses fracture Pterygoid processes	Yes-*LeFort*	I, the anterolateral margin of the nasal fossa	
		II, the inferior orbital rim	
		III, the zygomatic arch	
	No-	Medial: *Naso-orbito-ethmoidal complex,* Markowitz and Manson classification	type I -->medial canthal tendon is intact and connected to a single large fracture fragment.
			type II-->the fracture is comminuted, and the medial canthal tendon is attached to a single bone fragment.
			type III-→ comminution extends to the medial canthal tendon insertion site on the anterior medial orbital wall at the level of the lacrimal fossa, with resultant tendon avulsion.
		Lateral: *Zygomaticomaxillary complex*	
One/few buttresses fracture	*Mandible*→ characterized by location		
	*Orbital “blow out”* fracture		
	*Frontal sinus* fractures		
	*Alveolar process*		
	*Nasal bone*		

In midface fractures involving multiple buttresses and damage to the pterygoid plates, the three subtypes of Le Fort fractures should be considered. Determining whether the fracture predominantly affects the lateral or medial portion of the midface will help show whether the pattern corresponds to a fracture of the zygomaticomaxillary complex or naso-orbito-ethmoidal complex.

## Le Fort fractures

**Fig. 7:**
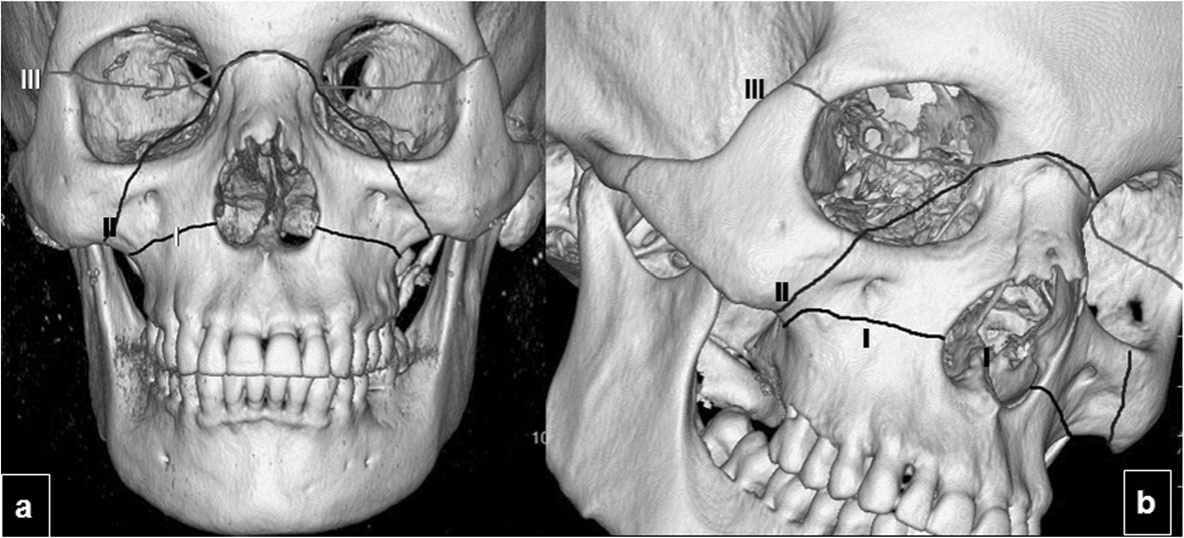
Le Fort fracture patterns. Three dimensional CT images of an adult skull in frontal (**a**) and oblique (**b**) orientations show the osseous facial structures classically affected in type I, II, and III Le Fort fractures

Le Fort fractures are complex facial fractures with varying degrees of craniofacial dissociation affecting various facial buttresses. In 1901, a French surgeon, René Le Fort, published the results of his experiments in which he applied blunt force to the midface of cadavers, finding three common patterns, all including a fracture through the pterygoid plates (Fig. 7) [21].

Although pterygoid plate fractures are often described in relation to Le Fort fractures, 37.3% of the patients with pterygoid plate fractures have craniofacial fracture patterns unrelated to Le Fort fractures [37].

The numerous components seen in Le Fort fractures make it difficult to classify these lesions. To simplify this task, Rhea et al. [38] proposed an algorithm that takes into account the fact that each of the Le Fort fractures has one or more components that are easily recognizable and unique to each: in Le Fort I fractures, the anterolateral margin of the nasal fossa; in Le Fort II fractures, the inferior orbital rim; and in Le Fort III fractures, the zygomatic arch (Table 2).

**Fig. 8:**
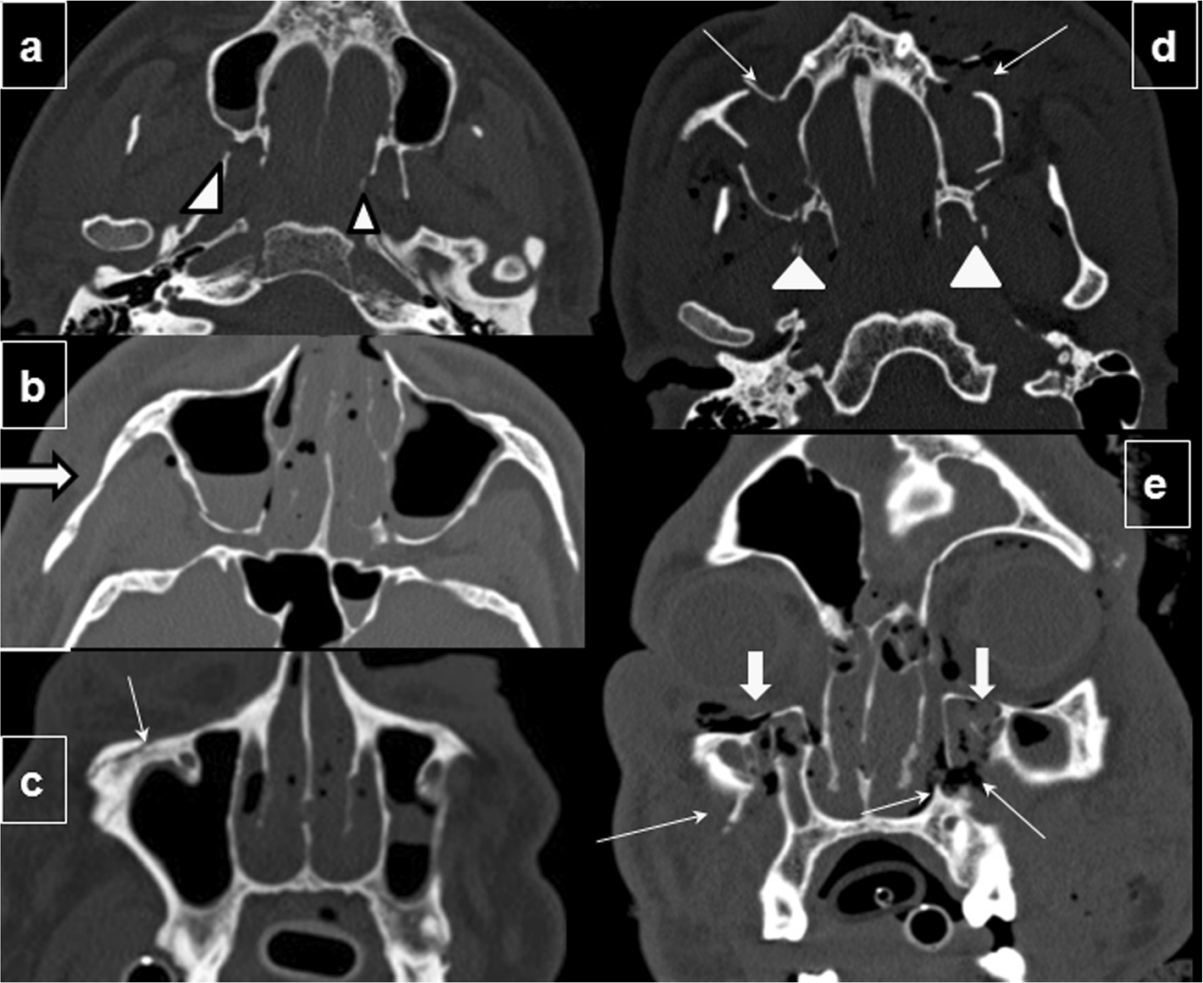
Two patients with multiple Le Fort fractures. **a**–**c** Patient one and (**d**, **e**) patient two. Patient one: **a** axial CT image at the level of the inferior maxillary sinus shows fractures in both pterygoid plates (arrowheads). **b** Axial image at the level of the inferior margin of the orbits shows zygomatic fractures on the right side, illustrating the definition of a Le Fort III fracture (thick arrow). **c** Coronal image shows involvement of the inferior orbital rim, illustrating the definition of a Le Fort type II fracture (thin arrow). Patient 2: **d** Axial CT image at an inferior level of the maxillary sinuses demonstrates bilateral fractures through the pterygoid plates (arrowheads) and maxillary sinus walls (arrows), findings indicative of Le Fort type I fractures. **e** Coronal CT image of the same patient shows a fracture of the inferior aspect of the maxillary sinus walls (thin arrows), a type I Le Fort fracture, and a fracture of the inferomedial orbital walls, a Le Fort type II fracture (thick arrows)

Depending on the way forces are distributed in the facial skeleton, Le Fort levels on the two sides of the face can be different, and fractures can occur through more than one Le Fort level on the same side of the face (Fig. 8), and they can also be incomplete. Fractures that are incomplete, that still have periosteal attachments intact in some areas or that are impacted, are the hardest to treat, so to report these data might be helpful for the surgeon.

Other facial fracture patterns such as naso-orbito-ethmoidal complex, frontal sinus, and zygomaticomaxillary complex fractures are often found together with Le Fort fractures. Fractures of the hard palate, maxillary dentoalveolar units, and mandible affect occlusion and thus require appropriate repair; so, it is important to check for these injuries specifically [30, 38].

Le Fort patterns are partially outdated in the first place because Le Fort’s experiments used low velocity trauma; higher velocity trauma more frequent nowadays results in different midface fracture patterns, although most cases can be described as variants of classical Le Fort fractures [39]. In second place, at present, there is good osteosynthesis hardware that can restore facial alignment hence the current emphasis on the subunits. The upper Le Fort level used to be important because there were few treatments that would not address the loss of anteroposterior projection eventually leading to elongated or flattened faces; however, the lower Le Fort level is still very important because of the occlusion, which can need early repair.

## Naso-orbito-ethmoid fractures

Injuries combining fractures of the nasal bone, medial orbital wall, and frontal process of the maxilla disrupt the naso-orbito-ethmoidal complex [30]. Fractures of the naso-orbito-ethmoidal complex occur when a high-power force impacts the nose anteriorly and is transmitted posteriorly through the ethmoid bone, resulting in severe comminution of both medial maxillary buttresses [37].

In fractures of the naso-orbito-ethmoidal complex, common complications include exophthalmos, telecanthus, and leakage of cerebrospinal fluid through the cribriform plate [37]. Other injuries such as nasofrontal duct injury and ocular injury are commonly associated [31].

**Fig. 9:**
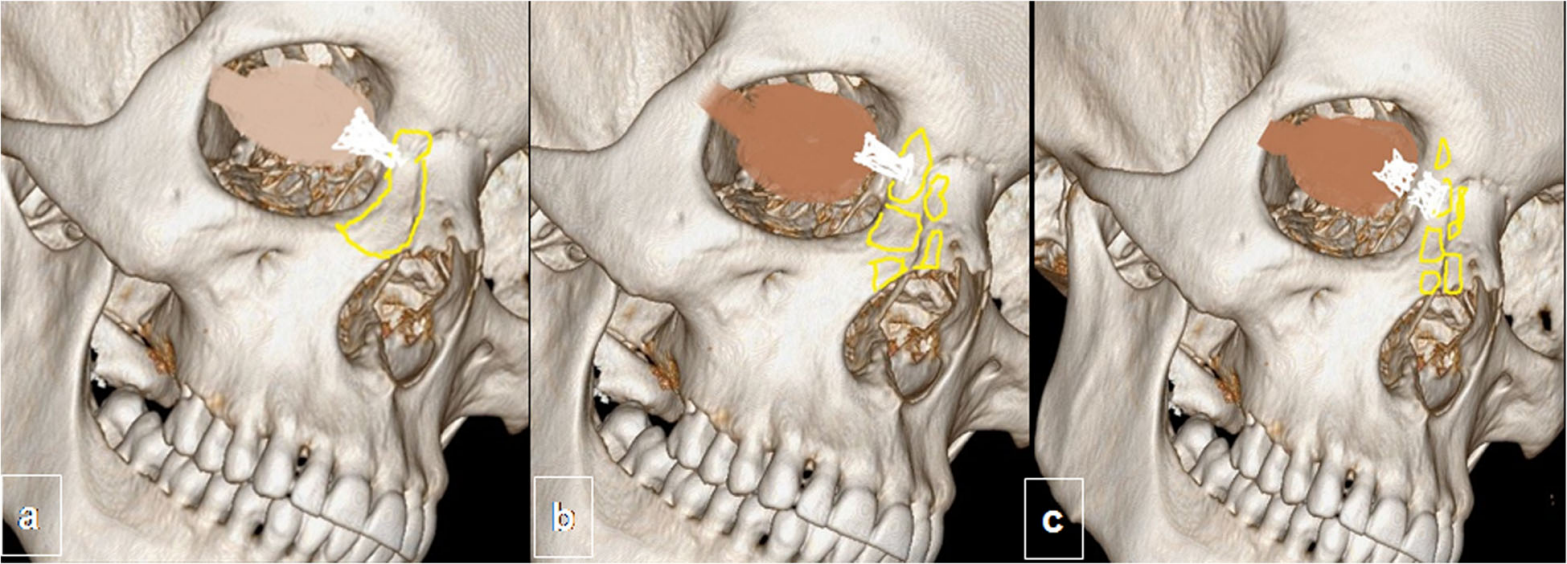
Classification of naso-orbito-ethmoidal fractures. Three dimensional CT images of an adult skull with color overlays depict the Markovitz and Manson classification: type I (**a**) characterized by a single large fragment with attached medial tendon; type II (**b**) with bone comminution without extension to the medial canthal tendon; and type III (**c**), where comminution affects the medial canthal tendon

**Fig. 10:**
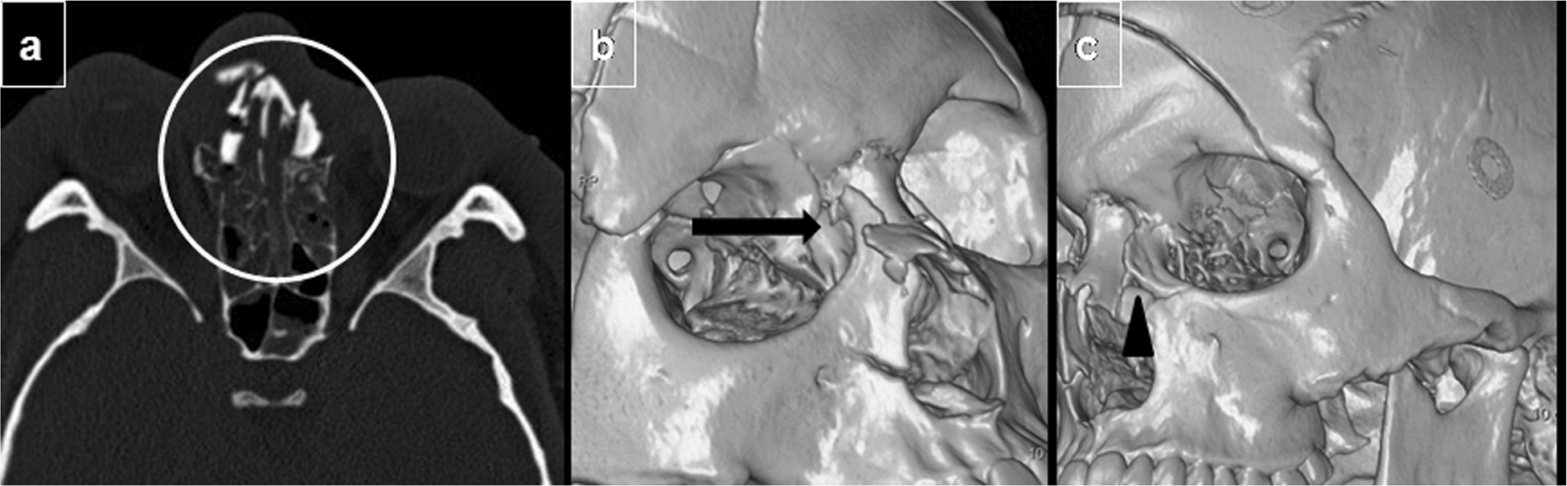
Bilateral naso-orbito-ethmoidal fracture. **a** Axial unenhanced CT image at the level of the mid-orbit shows a comminuted fracture of the naso-orbito-ethmoid complex (circled) resulting in telecanthus (widened intercanthal distance). **b**, **c** Oblique 3D reconstructions to assess the insertion of the medial canthal tendon on the bones of the lacrimal fossa: more significant comminution with an avulsed fragment can be seen on the right (arrow) than on the left side (arrowhead)

In the Markowitz and Manson classification system (Fig. 9), naso-orbito-ethmoidal complex fractures are classified by the extent of medial canthal tendon involvement (Table 2). In type I, an intact medial canthal tendon is attached to a single large fragment of fractured bone; in type II, the medial canthal tendon is attached to a single bone fragment of a comminuted fracture; whereas in type III, the medial canthal tendon is avulsed because comminution includes the tendon’s insertion site on the anterior medial orbital wall at the level of the lacrimal fossa (Fig. 10) [40].

The outer cardinal lines are composed of the frontal and zygomatic bone processes as well as the maxilla inferiorly. Generally, these outer lines are repaired first in complex injuries and provide the foundation upon which the more central naso-orbito-ethmoidal region can be reconstructed.

The most important point for classification is the displacement and/or comminution of the central fragment of the medial orbital wall, where the medial canthal tendon inserts [31]. Multidetector CT cannot depict the medial canthal tendon itself, but reporting the degree of comminution of the medial orbital wall at the level of the lacrimal fossa can help surgeons plan repair of the tendon [21].

It is also important to report the degree of comminution of the nasal bones, frontal processes of the maxilla, and nasal processes of the frontal bones to help surgeons decide whether bone grafting is required [30, 41].

## Zygomaticomaxillary complex fractures

**Fig. 11:**
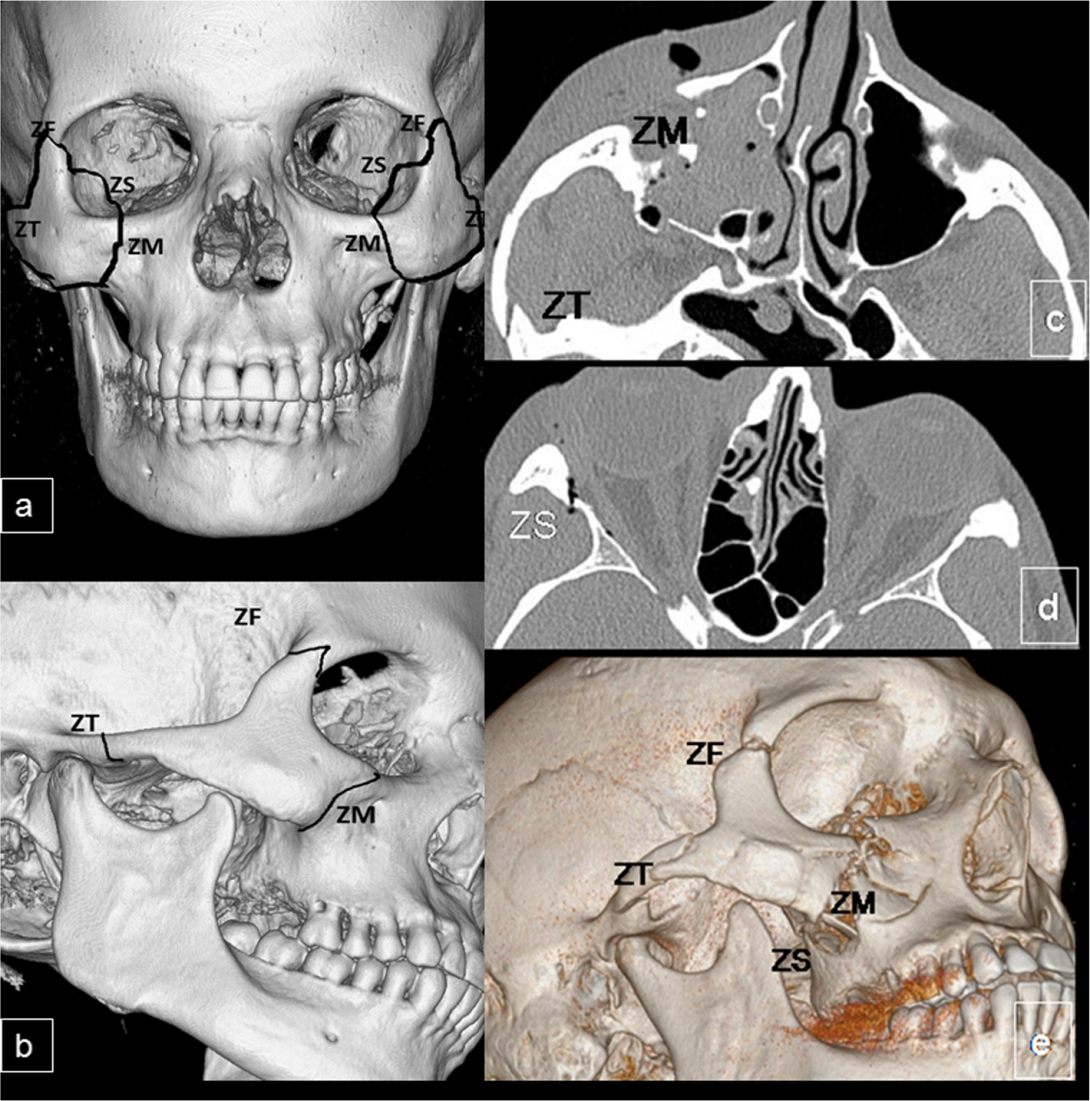
Zygomaticomaxillary complex. **a**, **b** Anatomy: three-dimensional CT image in frontal and lateral orientations shows the osseous anatomy of the zygomaticomaxillary complex: the zygomaticofrontal (ZF), zygomaticosphenoid (ZS), zygomaticomaxillary (ZM), and zygomaticotemporal (ZT) sutures. **c**–**e** Fracture of the zygomaticomaxillary complex. **c**, **d** Axial CT images show a nondisplaced fracture of zygomaticotemporal suture (ZT), comminution and angulation through the left zygomaticosphenoid (ZS), and significant displacement of the zygomaticomaxillary suture (ZM). **e** Three-dimensional CT images of the upper left facial region of the left ZMC fracture

A fracture of the zygomaticomaxillary complex results from a direct impact on the malar eminence that causes the underlying zygomatic bone to separate from the calvaria. The zygomatic bone is a paired irregular bone that forms part of the lateral orbital walls. Under normal conditions, it has four sutures with the rest of the facial skeleton and the calvaria. Zygomaticomaxillary complex fractures extend through these four borders (Fig. 11a, b). Also called a tetrapod or quadripod fracture, this injury used to be called a tripod fracture because plain-film radiography was only able to show three disrupted borders. In fact, the fracture extends posteriorly through the sphenozygomatic suture, so there are four elements [21].

The first and most important goal in treating zygomaticomaxillary complex fractures is to reduce the fracture. The zygomatic bone plays an important role in defining the height and width of the midface [42], so failure to recognize and treat this fracture can lead to cosmetic deformity. The complexity of the surgical repair will depend on the degree of fracture displacement and comminution [43], so the radiologist’s report should include these points (Fig. 11c–e).

The extent of orbital involvement will also determine surgical treatment because increased orbital volume is the most common cause of posttraumatic enophthalmos [30]. When more than 50% of the orbital floor is affected, open reconstruction will probably be necessary [43, 44].

Anyway, these fractures are different than simple orbital blow-out fractures, when considering the size of the floor fragment on pre-surgical CT: when ZMC fractures displaced with internal rotation/malar retrusion are reduced, the defect size must be taken together with the possibility that the defect will further expand with eventually a worsening of the future enophthalmos.

Finally, it is also important to take the status of the orbital apex into account. When the lateral orbital wall is displaced medially, apex involvement is more likely and consequently damage to carotid arteries and cranial nerves (CN) II–VI in the superior orbital fissure is more likely [45].

Zygomaticomaxillary complex fractures can be displaced due to rotational forces on the zygomatic bone from the masseter muscle; in these cases, if the infratemporal fossa is occupied, patients may have difficulties chewing [31].

## Mandible

**Fig. 12:**
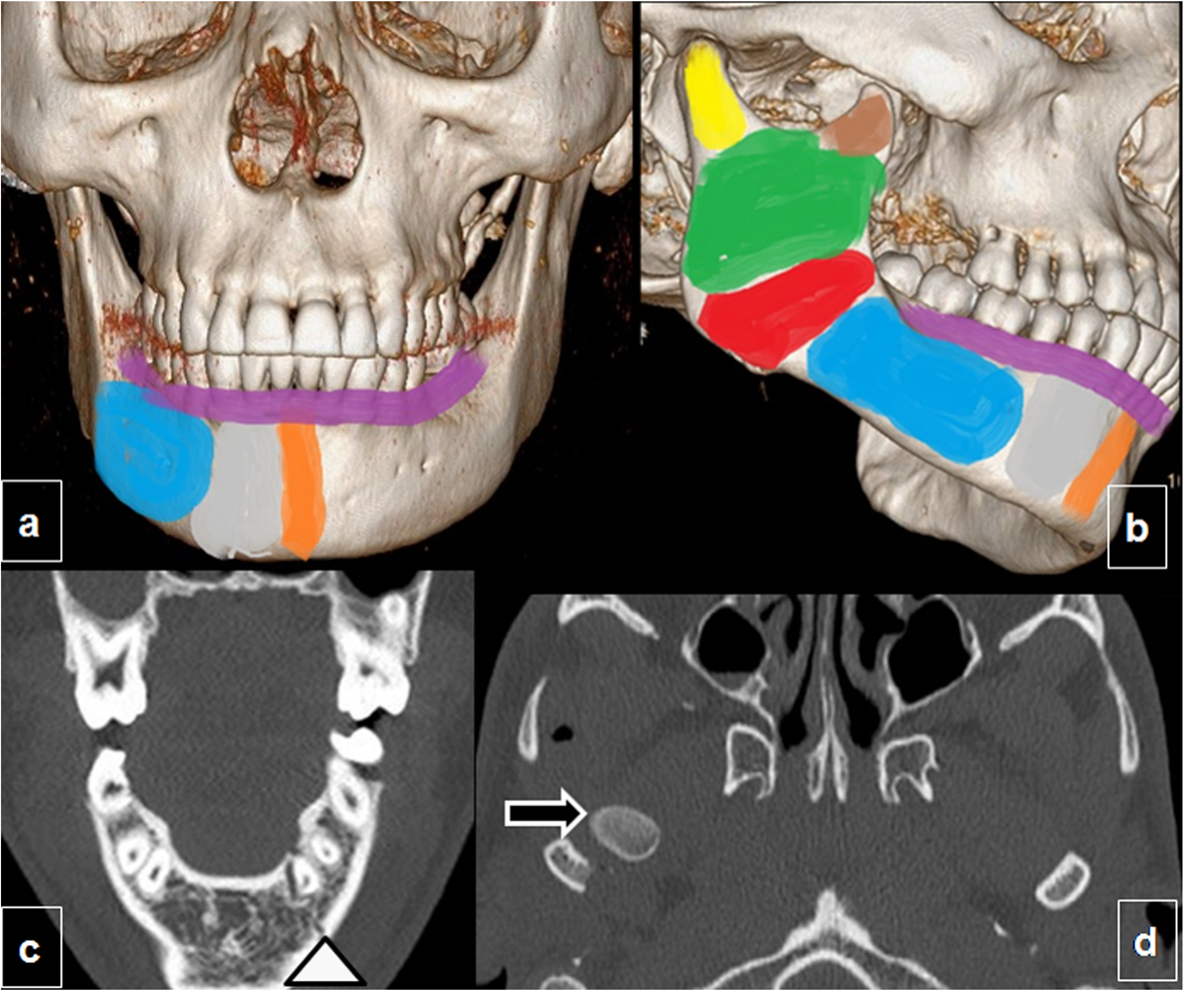
Fractures of the mandible. Anatomy (**a**, **b**): three-dimensional CT image with color overlays indicating the parts of the mandible in coronal and lateral oblique orientations: the alveolar process (purple), symphysis (orange), parasymphyseal region (gray), body (blue), angle (red), ramus (green), coronoid process (brown), and condyle (yellow). **c**, **d** Bifocal mandibular fracture. **c** Coronal and (**d**) axial unenhanced CT of the mandible shows the classical pattern of bifocal mandibular fractures, affecting the body of the mandible on one side (arrowhead in **c**) and the contralateral condyle with accompanying luxation (arrow in **d**)

After the nasal bones, the mandible is the most common site of facial fractures; mandibular fractures often require open reduction. Plain-film panoramic radiography has been supplanted by CT as the first-line examination because of its availability in trauma settings. Fractures are characterized according to their location (Fig. 12a, b), the degree of comminution, and the presence of displaced fragments.

The mandible is a U-shaped bone that is connected to the calvaria through the temporomandibular joints, creating a ring-like structure. This ring-like configuration means that an impact on the mandible usually results in two or more separate fractures. When a single fracture of the mandible is visualized, it is usually due to a fracture-dislocation complex in which the temporomandibular joints have been relocated [31] (Fig. 12).

When a mandibular fracture results in three or more fragments within the same anatomic region, it is considered comminuted; when five or more fragments are present, it is considered severely comminuted [46]. Severely fragmented bone that has lost its periosteal attachment is likely to become devitalized and is typically removed. Mandibular fractures are often triangular basal fracture segments, sometimes called “basal triangles,” that can be seen anywhere along the inferior border of the mandible [22, 46].

When the mandibular canal is involved, the inferior alveolar nerve may be damaged, resulting in a loss of sensation, especially if displacement exceeds 5 mm [22, 31, 47].

## Orbital wall “blowout”

**Fig. 13:**
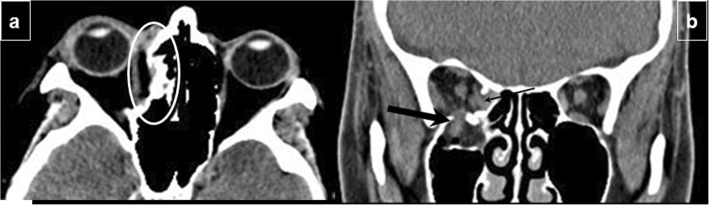
Orbital blowout. **a** Axial CT image shows disruption of the internal wall of the orbit with fat herniation (circled). **b** Coronal view shows internal (thin arrow) and inferior (thick arrow) wall disruption; the inferior rectus muscle has herniated and has consequently lost its normal flattened appearance

There are two main types of orbital fractures: those that form part of a larger fracture pattern (zygomaticomaxillary complex, naso-orbito-ethmoidal complex, or Le Fort fractures) and isolated blowout fractures. The first type occurs when one or more of the bony walls of the orbit are fractured in particular in the setting of a larger fracture; the inferior orbital rim is the most commonly affected part. The second type, known as orbital “blowout” fracture, occurs when direct traumatic impact on the globe is transmitted to the orbital roof, floor, or medial wall, displacing it outward, away from the orbit (Fig. 13), while the orbital rim itself remains intact [30]. Blowout fractures most often affect the inferior part of the orbital wall, followed by the medial part [31]; when one fractured wall is detected, radiologists should look carefully at the other walls [30].

Two other types of orbital wall fractures are orbital roof fractures and pediatric “trapdoor” fractures. The roof is the only part of the orbital wall that separates the anterior cranial fossa from the contents of the orbit; injury to this structure can result in a dural tear and consequently a cerebrospinal fluid leakage or a brain herniation. The trapdoor fracture is a type of orbital blowout fracture affecting the inferior part of the orbital wall, with the particularity that the inferior rectus muscle bulges into the maxillary sinus and is entrapped when the fractured fragment returns to its original position. Trapdoor fractures typically occur in children. Coronal multidetector CT images show the inferior rectus muscle below the orbital floor, sometimes also showing the fractured fragment of the inferior orbital wall [31].

Isolated orbital fractures are treated for one of three reasons: to free entrapped extraocular muscles, to prevent postoperative malpositioning and resulting complications (diplopia or enophthalmos) in large fractures, and to decompress neural structures in very severe cases where the lateral wall protrudes into the orbital apex or middle cranial fossa. Muscle entrapment (infrequent in adults due to comminution of the floor) is a surgical emergency, so surgeons should be notified immediately when herniation is identified on CT; it is very useful to inform surgeons of the approximate size of the fractures and the degree of displacement of fat and soft tissues [48–51]. Other complications include intraorbital hemorrhage, globe injury, and infraorbital nerve injury in cases of orbital floor fracture [31].

## Frontal sinus fractures

In fractures of the upper third of the face, the wall of the frontal sinus is usually involved because this is the part of the frontal bone that is the least thick [31]. These fractures are classified according to whether the anterior wall, posterior wall, or both are involved and according to the degree of displacement and comminution of the fracture [30].

**Fig. 14:**
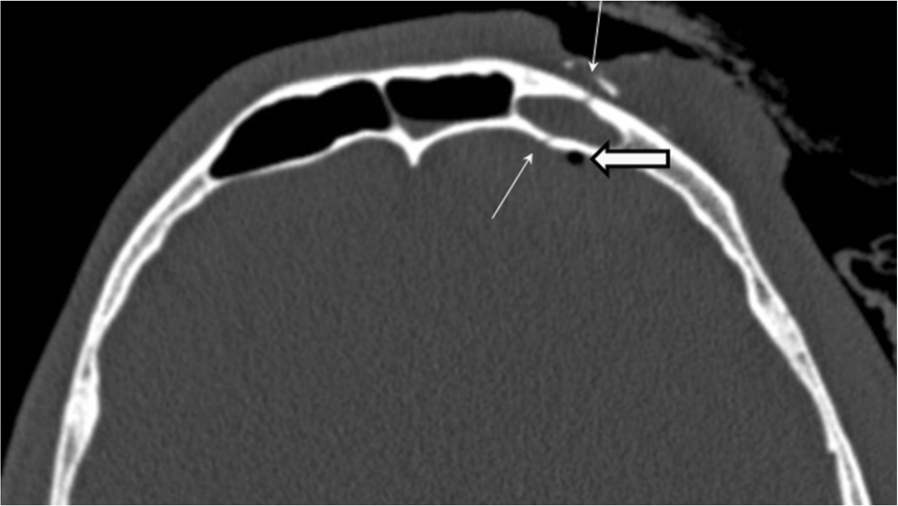
Frontal sinus fracture. Axial unenhanced CT image of the frontal bone demonstrates a nondisplaced fracture of the anterior and posterior walls (thin arrows) of the left frontal sinus, with partial opacification of the frontal sinus and small foci of pneumocephalus (thick arrow)

In posterior wall fractures, it is paramount to report whether pneumocephalus is present and the degree of bone loss in the posterior wall and floor of the sinus, because these findings will help the surgeon gauge the probability of anterior cranial fossa involvement. In these cases, brain injury is often associated (Fig. 14) [52, 53]. When the fracture extends to the posterior wall, it creates a communication that connects the frontal sinus with the anterior cranial fossa, increasing the likelihood of complications such as cerebrospinal fluid leakage and rhinorrhea, brain herniation, and intracranial infection. When the fracture occurs along the medial aspect of the frontal sinus and extends into the nasofrontal duct, it may cause a mucocele that obstructs the drainage of the sinus; this blockage can result in superinfection extending into the bone or even into the brain [52–56].

It is important to examine this area carefully. A recent study found that a significant percentage of fractures of the anterior skull base, cribriform plate, or sella turcica were missed in reports done during calls [57].

## Alveolar process

**Fig. 15:**
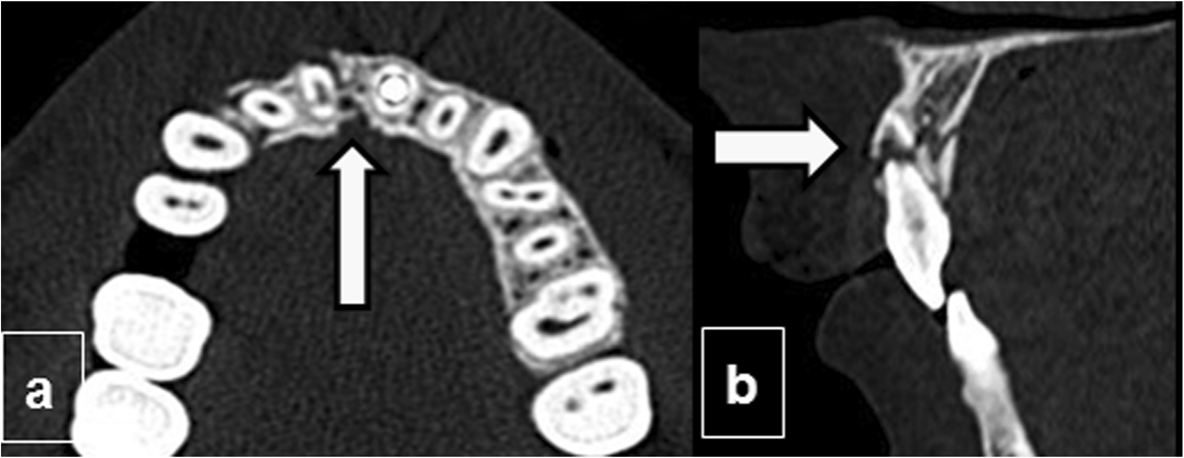
Alveolar process fracture. Axial (**a**) and sagittal (**b**) unenhanced CT images show a fracture of the upper alveolar ridge with associated dental root fracture (arrows)

Alveolar process fractures are the most commonly observed pattern of maxillary fracture. Caused by direct force on the alveolar process or by indirect force from an impact on the teeth below through the base of the dental crown, these fractures must be treated with surgical debridement and prophylactic antibiotics to avoid bacterial infection from the oral cavity [31]. Alveolar process fractures involve a risk of dental root avulsion, crown or root fracture (Fig. 15), dental intrusion or extrusion, and malocclusion [58]. A tooth can also be aspirated into the airway, leading to pulmonary atelectasis.

## Nasal bone

The superficial location of the nose and the relative thinness of the nasal bones mean that nasal bone fractures are the most common injuries to the facial skeleton [31]. The two nasal bones articulate with the frontal bone at the frontonasal suture and with the frontal process of the maxilla at the nasomaxillary suture, forming the bony nasal pyramid. It can be easy to miss subtle fractures of the pyramidal bone, anterior nasal spine, and bony septal fractures. If any of these injuries are detected, the septal cartilage must be examined with a speculum [36].When fracture the nasal cartilage, they can disrupt the perichondrium, causing a septal hematoma, which can lead to various complications such as impairment of nasal airflow, abscess formation, and necrosis that can even perforate the septum.

**Fig. 16:**
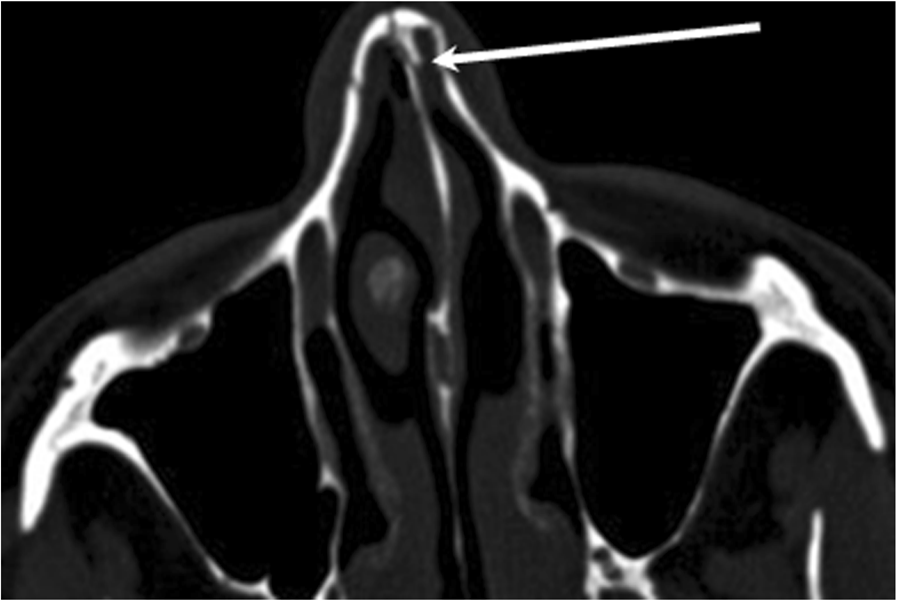
Nasal bone fracture. Axial unenhanced CT image shows nondisplaced nasal bone fractures extending to the anterior bone septum (arrow)

Nasal bone fractures are classified according to the anatomic plane involved. Type 1 fractures affect only the region below the plane that extends from the caudal tip of the nose to the anterior nasal spine; the nasal septum is unaffected in these fractures. By contrast, in type 2 fractures, both the septum and the anterior nasal spine are involved (Fig. 16). Finally, type 3 fractures involve the bones that surround the orbit and sometimes intracranial structures in addition to the nasal bone and septum [31].

In low-force impacts, trauma often causes isolated fracture of the nasal bones. However, because the nasal bones are located close to the ethmoid sinuses and the medial orbital walls, high-force impacts can also cause injury to the underlying ethmoid sinuses and orbit. The close physical and functional relationships among the bony structures in this area have led some authors to recommend that the nasal-orbital-ethmoid region be considered a single unit in cases of high-impact trauma [30]. Other authors stress the importance of separating simple nasal fractures from more serious naso-orbito-ethmoid fractures that extend into the nose through the ethmoid bones [59].

## Conclusions

It is essential to use shared terminology to refer to the pattern of facial fractures in radiology reports. Descriptors such as naso-orbito-ethmoidal complex, zygomaticomaxillary complex, and orbital “blowout” can be extremely useful for surgeons, so they should be used when possible. Surgeons require information about the anatomic landmarks and features of the fracture such as the degree of displacement and comminution so they can plan treatment and predict possible complications.

## Data Availability

Data sharing is not applicable to this article as no datasets were generated or analyzed during the current study.

## Abbreviation

CN: Cranial nerve
NLD: Nasolacrimal duct
